# A Bayesian inference method for the analysis of transcriptional regulatory networks in metagenomic data

**DOI:** 10.1186/s13015-016-0082-8

**Published:** 2016-07-08

**Authors:** Elizabeth T. Hobbs, Talmo Pereira, Patrick K. O’Neill, Ivan Erill

**Affiliations:** Department of Biological Sciences, University of Maryland Baltimore County (UMBC), 1000 Hilltop Circle, Baltimore, MD 21250 USA

**Keywords:** Transcription factor, Regulatory network, Regulon, Metagenomics, Bayesian inference, Copper homeostasis, Metal resistance, Stress response, CsoR

## Abstract

**Background:**

Metagenomics enables the analysis of bacterial population composition and the study of emergent population features, such as shared metabolic pathways. Recently, we have shown that metagenomics datasets can be leveraged to characterize population-wide transcriptional regulatory networks, or meta-regulons, providing insights into how bacterial populations respond collectively to specific triggers. Here we formalize a Bayesian inference framework to analyze the composition of transcriptional regulatory networks in metagenomes by determining the probability of regulation of orthologous gene sequences. We assess the performance of this approach on synthetic datasets and we validate it by analyzing the copper-homeostasis network of Firmicutes species in the human gut microbiome.

**Results:**

Assessment on synthetic datasets shows that our method provides a robust and interpretable metric for assessing putative regulation by a transcription factor on sets of promoter sequences mapping to an orthologous gene cluster. The inference framework integrates the regulatory contribution of secondary sites and can discern false positives arising from multiple instances of a clonal sequence. Posterior probabilities for orthologous gene clusters decline sharply when less than 20 % of mapped promoters have binding sites, but we introduce a sensitivity adjustment procedure to speed up computation that enhances regulation assessment in heterogeneous ortholog clusters. Analysis of the copper-homeostasis regulon governed by CsoR in the human gut microbiome Firmicutes reveals that CsoR controls itself and copper-translocating P-type ATPases, but not CopZ-type copper chaperones. Our analysis also indicates that CsoR frequently targets promoters with dual CsoR-binding sites, suggesting that it exploits higher-order binding conformations to fine-tune its activity.

**Conclusions:**

We introduce and validate a method for the analysis of transcriptional regulatory networks from metagenomic data that enables inference of meta-regulons in a systematic and interpretable way. Validation of this method on the CsoR meta-regulon of gut microbiome Firmicutes illustrates the usefulness of the approach, revealing novel properties of the copper-homeostasis network in poorly characterized bacterial species and putting forward evidence of new mechanisms of DNA binding for this transcriptional regulator. Our approach will enable the comparative analysis of regulatory networks across metagenomes, yielding novel insights into the evolution of transcriptional regulatory networks.

**Electronic supplementary material:**

The online version of this article (doi:10.1186/s13015-016-0082-8) contains supplementary material, which is available to authorized users.

## Background

The advent of next-generation sequencing methodologies has enabled the study of bacterial populations through direct sampling of their genetic material [1]. Metagenomics techniques allow the detailed investigation of bacterial communities, their shared metabolic pathways and their interaction with environment and hosts [2–7], but they also pose many challenges regarding data standardization, processing and analysis [8, 9]. To date, most analyses of metagenomics datasets have focused on the phylogenetic composition of metagenomes and the relative contribution of different bacterial clades to metabolic pathways [3, 9–12]. However, metagenomics data also constitute a powerful resource for the direct analysis of transcriptional regulatory networks, or regulons, in natural environments. Such analyses can be used to characterize the contribution of non-culturable bacteria and mobile genetic elements to global regulatory networks, to analyze the changes in a population’s regulatory program in response to interventions or habitat adaptation, and to quantify the relative importance of genetic elements in the makeup of known regulatory systems. Comparative research on multiple metagenomes has revealed that regulatory potential, measured as the local density of putative transcription factor (TF)-binding sites, correlates with processes involved in the response to stimuli present in specific environments [13, 14]. Recently, we provided proof of concept that TF-binding motifs can be effectively leveraged to analyze the genetic makeup of known transcriptional regulatory networks using metagenomic data, providing insights into the function of such networks in specific microbiomes [15]. In this work we formalize an inference method to analyze transcriptional regulatory networks in metagenomics datasets. The Bayesian inference approach we put forward provides a consistent framework for the study of regulatory networks using metagenomics datasets, facilitating the interpretation of results, standardizing the outcome of analyses to facilitate comparison and allowing users to selectively adjust sensitivity. We validate the novel inference framework on the Integrated Reference Catalog of the Human Gut Microbiome [16], analyzing the regulation of copper-homeostasis in gut microbiome Firmicutes through the recently characterized copper-responsive repressor CsoR [17]. Our results reveal an inferred copper-homeostasis network congruent with that reported in studies on model organisms, outlining the core elements of this regulatory system and highlighting specific features of the human gut CsoR meta-regulon.

## Methods

### Datasets

Human gut metagenomics data was obtained from the Integrated Reference Catalog of the Human Gut Microbiome service (http://meta.genomics.cn/) [16]. The dataset contains 1267 gut metagenomes, totaling 6.4 Tb. To ensure consistency, here we restricted the analysis to 401 samples from healthy European individuals obtained in the MetaHIT project. This subset contains 5,133,816 predicted genes, with roughly half of them (2,579,737) functionally annotated with eggNOG/COG identifiers from the eggNOG v4.0 database [18]. The bacterial population in these 401 samples is dominated by two bacterial orders [Bacteroidales (58.51 %) and Clostridiales (32.11 %)] belonging to two major bacterial phyla [Bacteroidetes (59.29 %) and Firmicutes (34.97%)]. A CsoR-binding motif was compiled by combining experimentally-validated and computationally inferred Firmicutes CsoR-binding sites available in the CollecTF and RegPrecise databases [19, 20].

### Data processing

For each sample and scafftig therein, predicted open-reading frames (ORF) in the same strand and with a conservative intergenic distance (<50 bp) were considered to constitute an operon. Only operons with a complete lead ORF (containing a predicted translational start codon on their 5′ end) and at least 60 bp of sequence upstream of the translational start codon were considered for analysis. We also excluded from analysis any operons with no gene product mapping to a Firmicutes reference genome [15]. Taxonomical and eggNOG information for all ORFs in the remaining 752,783 operons was re-annotated by searching the eggNOG v4.0 database with DIAMOND [21]. The available upstream region (up to 300 bp) for these operons was scored on both strands with the position-specific scoring matrix (PSSM) derived from the compiled CsoR-binding motif using a Laplacian pseudocount and equiprobable background base frequencies [22]. For every sequence position, the scores from both strands were combined following the soft-max function (Additional file 1):

1 $$PSSM(S_{i} ) = \log_{2} \left( {2^{{PSSM(S_{i}^{f} )}} + 2^{{PSSM(S_{i}^{r} )}} } \right)$$

where* PSSM*(*S*_*i*_) denotes the combined PSSM score of a site at position *i* and *PSSM*(*S*_*i*_^*f*^) and *PSSM*(*S*_*i*_^*r*^) denote the score of the site at position *i* in the forward and reverse strands, respectively.

### Inference method

For a given eggNOG/COG functional identifier, we consider the set of promoters (*D*) from all operons containing at least one gene mapping to that eggNOG/COG. We define two theoretical distributions for the set of positional PSSM scores in promoters associated with a particular eggNOG/COG identifier. If the eggNOG/COG is not regulated by the TF, we expect that the promoters mapping to it display a background distribution of scores (*B*), which we can approximate by a normal distribution parametrized by the statistics of the set of all promoters in the metagenome (*G*):

2 $$B\sim N(\mu_{g} ,\sigma_{g}^{2} )$$

Conversely, for an eggNOG/COG regulated by the TF, the distribution of PSSM scores (*R*) in promoters should be a mixture of the background distribution and the distribution of scores in functional sites. Again, we can approximate the distribution of scores in functional sites with a normal distribution parametrized by the statistics of the known sites belonging to the TF-binding motif (*M*).

3 $$R\sim \alpha N(\mu_{m} ,\sigma_{m}^{2} ) + (1 - \alpha )N(\mu_{g} ,\sigma_{g}^{2} )$$

The mixing parameter *α* corresponds to the probability of observing a functional binding site in a regulated promoter, which can be estimated from known instances of TF-binding sites in their genomic context. For CsoR, we expect on average one binding site in a regulated promoter of length 300 bp, so *α* is defined to be 1/300 [23, 24].

Given a promoter* D*_*i*_ from the set of promoters (*D*) mapping to a particular eggNOG/COG identifier, we seek to obtain the probability that the eggNOG/COG is regulated by the TF. Formally, we seek to obtain the posterior probability of the mixture distribution of scores (*R*) given the scores *s*_*j*_ observed in the promoter mapping to the eggNOG/COG (*D*_*i*_):

4 $$P(R|D_{i} ) = \frac{{P(D_{i} |R)P(R)}}{{P(D_{i} )}}$$

After applying the law of total probability, we can express this more conveniently in a likelihood ratio form:

5 $$P(R|D_{i} ) = \frac{{P(D_{i} |R)P(R)}}{{P(D_{i} |R)P(R) + P(D_{i} |B)P(B)}} = \frac{1}{{1 + \frac{{P(D_{i} |B)P(B)}}{{P(D_{i} |R)P(R)}}}}$$

The likelihood functions *P*(*D*_*i*_*|R*) and *P*(*D*_*i*_*|B*) can be estimated for a given score *s*_*j*_ using the density function of the *R* and *B* distributions defined above. If we assume approximate independence among the scores at different positions, we obtain:

6 $$P(D_{i} |R) = \prod\limits_{{s_{j} \in D_{i} }} {L\left( {s_{j} |\alpha N(\mu_{m} ,\sigma_{m}^{2} ) + (1 - \alpha )N(\mu_{g} ,\sigma_{g}^{2} )} \right)}$$

and

7 $$P(D_{i} |B) = \prod\limits_{{s_{j} \in D}} {L\left( {s_{j} |N(\mu_{g} ,\sigma_{g}^{2} )} \right)}$$

The priors* P(R)* and* P(B)* can be inferred from genomic data.* P(R)* and* P(B)* can be approximated by the fraction of annotated operons in a genome that are known and not known, respectively, to be regulated by the TF. Using *B. subtilis* as a reference genome for CsoR, we obtain* P(R)* = 3/1811 and* P(B)* = 1 − *P(R)*.

The contributions of all promoters *D*_*i*_ mapping to a particular eggNOG/COG can be assumed to be independent. Therefore, we obtain:

8 $$P(R|D) = \frac{1}{{1 + \left( {\prod\nolimits_{{D_{i} \in D}} {\frac{{P(D_{i} |B)}}{{P(D_{i} |R)}}} } \right)\frac{P(B)}{P(R)}}}$$

where we can naturally assign a likelihood ratio product of 1 to any eggNOG/COGs that presents no mapped promoters in the samples under analysis.

### Sensitivity adjustment and determination of putatively regulated eggNOG/COGs

The large size of metagenomics datasets poses challenges for the efficient computation of the posterior probabilities outlined above. It is known that a large fraction of the eggNOG/COG identifiers will not be regulated by the TF. The computation may therefore be simplified by defining a score threshold to exclude operons with promoters that show no evidence of regulation [15]. This strategy has the added benefit of compensating for heterogeneity in eggNOG/COG clustering, which may assign distant orthologs to the same eggNOG/COG identifier, potentially diluting the contribution of a regulated ortholog to the eggNOG/COG posterior probability.

Formally, we consider the subset of the promoters D^*^⊂ *D* mapping to a particular eggNOG/COG that have at least one score above a predefined threshold *θ*. That is, *D*_*i*_∈ D^*^ if max(*s*_*j*_∈ *D*_*i*_) ≥ *θ*. It follows that we should adjust the score likelihoods of Eqs. 6 and 7 to take into account the fraction of probability mass assigned to the data that will not be observed in the reduced promoter set D^*^. The probability of observing a promoter* D*_*i*_ with no positions *p*_*j*_ scoring above the threshold* θ* under the background (*B*) and regulated (*R*) models is given by the cumulative distribution function (*Φ*) for each model:

9 $$U_{B} = \prod\limits_{{p_{j} \in D_{i} }} {\left( {\Phi (\theta ,\mu_{g} ,\sigma_{g}^{2} )} \right)}$$

10 $$U_{R} = \prod\limits_{{p_{j} \in D_{i} }} {\left( {\alpha \Phi (\theta ,\mu_{m} ,\sigma_{m}^{2} ) + (1 - \alpha )\Phi (\theta ,\mu_{g} ,\sigma_{g}^{2} )} \right)}$$

Hence, the probability of observing a promoter with at least one score above the threshold* θ* under the background (*B*) and regulated (*R*) models is given by (1 − *U*_*B*_) and (1 − *U*_*R*_), respectively. We can use these probabilities to normalize the likelihoods as follows:

11 $$P(D_{i} |R) = \frac{{\prod\nolimits_{{s_{j} \in D_{i} }} {L\left( {s_{j} |\alpha N(\mu_{m} ,\sigma_{m}^{2} ) + (1 - \alpha )N(\mu_{g} ,\sigma_{g}^{2} )} \right)} }}{{(1 - U_{R} )}}$$

12 $$P(D_{i} |B) = \frac{{\prod\nolimits_{{s_{j} \in D_{i} }} {L\left( {s_{j} |N(\mu_{g} ,\sigma_{g}^{2} )} \right)} }}{{(1 - U_{B} )}}$$

Similarly, the priors *P(R)* and *P(B)* must be renormalized by multiplying the observed number of regulated and non-regulated operons in a reference genome by (*1 − U*_*B*_) and (*1 − U*_*R*_), respectively, in order to account for the fact that thresholding alters the base rate at which regulated promoters are observed.

The inference method outlined above assigns a posterior probability value* P(D|R)* to all eggNOG/COG identifiers present in the metagenome. Ultimately, however, we wish to extract a set of putatively regulated eggNOG/COG for further analysis. This requires discretization of the list of posterior probabilities. Formally, given a list of eggNOG/COGs *S* with posterior probabilities $\vec{p}$, we wish to find a sublist S^*^ with posterior probabilities $\vec{p}^{*}$, so that the mean probability of regulation for a promoter chosen uniformly at random from S^*^ is at least (*1−φ*). To define S^*^, let $\vec{p}$ be sorted in reverse order and *S* be sorted similarly. Then let *n* be the greatest integer such that:

13 $$\frac{1}{n}\sum\limits_{i = 0}^{n} {p_{i} } \le (1 - \phi )$$

and set S^*^ = {*S*_*0*_,…,*S*_*n*_}. S^*^ is therefore the largest sublist of *S* having average posterior probability of at least (1−*φ*).

### Permutation test

Several alternative methods can be proposed to determine putatively regulated eggNOG/COGs in a metagenomic dataset. To benchmark the Bayesian framework introduced above against a frequentist approach, we define a permutation test based on the likelihood function *P*(*D*_*i*_*|R*) of Eq. 6. Given the original TF-binding motif defined by the collection of TF-binding sites, we generate *F* random symmetrical permutations of the TF-binding motif and parametrize their score distribution under the background (*B*_*f*_) and regulated (*R*_*f*_) models following Eqs. 2 and 3. Hence, for each permuted model *f*, we can compute the likelihood of the score distribution observed in a given promoter (*D*_*i*_) as:

14 $$P(D_{i} |R_{f} ) = \prod\limits_{{s_{j} \in D_{i} }} {L\left( {s_{j} |\alpha N(\mu_{{m^{f} }} ,\sigma_{{m^{f} }}^{2} ) + (1 - \alpha )N(\mu_{{g^{f} }} ,\sigma_{{g^{f} }}^{2} )} \right)}$$

Under the approximation of independence between promoter sequences used in Eq. 8, we can define *P(D|R)* for an eggNOG/COG as follows:

15 $$P(D|R_{f} ) = \prod\limits_{{D_{i} \in D}} {P(D_{i} |R_{f} )}$$

For each eggNOG/COG, we then can empirically approximate the p-value as the probability of obtaining a score distribution as extreme as the one observed in the promoters mapping to an eggNOG/COG given the null hypothesis that the distribution of scores is due to chance:

16 $$p = P\left( {P(D|R_{f} ) \ge P(D|R)} \right) \approx \frac{{1 + \sum\nolimits_{f = 1}^{F} {I\left( {P(D|R_{f} ) \ge P(D|R)} \right)} }}{F + 1}$$

where *I(·)* is the indicator function.

The permutation test therefore defines an alternative statistic to assess putative regulation of an eggNOG/COG based on the distribution of scores in the promoters mapping to it.

## Results

### Validation of the Bayesian inference pipeline on synthetic datasets

**Fig. 1:**
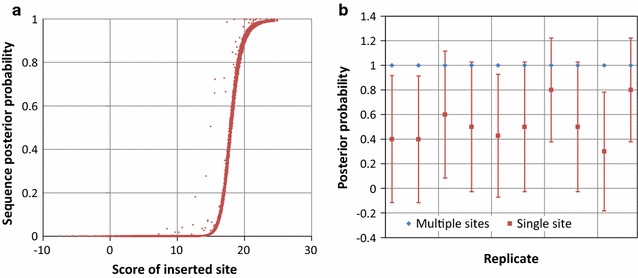
**a** Posterior probability of a 300 bp-long randomly generated sequence (40 % G + C) as a function of the score of a sampled CsoR site inserted at the first position of the sequence. The *plot* shows the results of 10,000 independent replicates. **b** Average posterior probability of a simulated eggNOG/COG. The eggNOG/COG contains 100 (300 bp-long, 40 % G + C) sequences, 30 of which contain inserted sites. Sites were either sampled randomly from the CsoR motif and inserted the first 30 sequences (multiple sites) or a single site was sampled from the motif and inserted in the first 30 sequences (single site). The *plot* shows the results of 10 independent experiments for each case. *Vertical bars* denote the standard deviation

To assess the behavior of the proposed inference framework, we evaluated its performance on synthetic datasets consisting of randomly generated sequence backgrounds with inserted sites sampled from the CsoR motif. Figure 1a shows the posterior probability *P(R|D)* of individual sequences (Eq. 5) as a function of the score of the inserted CsoR sites. The observed upward deviations from the baseline sigmoidal shape illustrate the ability of the inference method to integrate contributions from secondary sites, which occur at a low frequency in randomly generated sequences. Figure 1b compares the behavior of the posterior probability for an eggNOG/COG (Eq. 8) between a simulated eggNOG/COG in which sequences contain sites randomly sampled from the CsoR motif distribution and an eggNOG/COG in which the sequences containing sites are clonal. Multiple instances of a clonal sequence containing a putative TF-binding site are often found in metagenome samples. On average, the method assigns lower posterior probabilities to clonal sequences, hence decreasing the likelihood of designating the corresponding eggNOG/COG as putatively regulated.

**Fig. 2:**
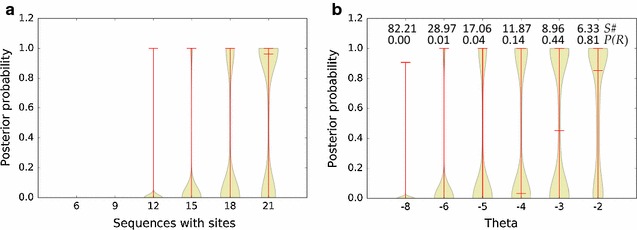
**a** Posterior probability of simulated eggNOG/COGs containing 100 (300 bp-long, 40 % G + C) randomly generated sequences, with 6, 9, 12, 15, 18 and 21 of them containing inserted sites sampled randomly from the CsoR motif. The *plot* shows the distribution of posterior probability for the eggNOG/COGs in 1000 simulated replicates as a function of the number of inserted sites. *Vertical bars* denote the standard deviation, with a *horizontal bar* indicating the median. **b** Sensitivity adjusted posterior probability of simulated eggNOG/COGs containing 100 (300 bp-long, 40 % G + C) randomly generated sequences, 12 of which contain inserted sites sampled randomly from the CsoR motif. The *plot* shows the distribution of posterior probability for the eggNOG/COGs adjusted for sensitivity in 1000 simulated replicates as a function of the sensitivity threshold *θ*, expressed as the number of standard deviations below the motif mean score. *Vertical bars* denote the standard deviation, with a *horizontal bar* indicating the median. The legends on* top* indicate the average number of sequences selected for analysis (*S#*) and the adjusted prior for regulation *P(R)* for each sensitivity threshold

**Fig. 3:**
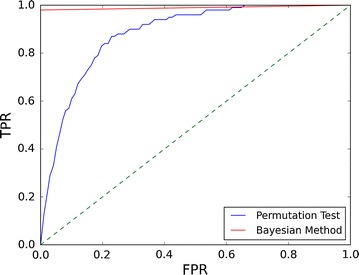
Receiver-operating characteristic (ROC) curve using the Bayesian posterior probability (Eq. 8) and the permutation test p-value (Eq. 15) as predictors of eggNOG/COG regulation. The ROC was generated on a synthetic dataset of 10,000 eggNOG/COGs, each with 100 promoter sequences mapping to it. To compute p-values, 100 permuted models were generated. The synthetic dataset contained 100 “regulated” eggNOG/COGs. To simulate real conditions, promoters mapping to “regulated” eggNOG/COGs were assigned sites following the CsoR motif based on a geometric distribution with an expectation of 0.33 sites per promoter

Figure 2a documents the behavior of the eggNOG/COG posterior probability (Eq. 8) as a function of the number of sequences with functional sites mapping to the eggNOG/COG. The results show that when the proportion of sequences containing functional sites among those mapping to an eggNOG/COG falls below 20 %, the posterior probability decreases sharply. Figure 2b illustrates the effect of introducing the sensitivity adjustment outlined in Eqs. 11 and 12. In addition to speeding up the computation, the use of a score threshold *θ* to exclude sequences with no evidence of regulation makes it possible to obtain high posterior probability values for eggNOG/COGs with less than 20 % sequences containing functional sites. This allows detecting putative regulation in heterogeneous eggNOG/COGs where the regulated ortholog is a minority contributor. In Fig. 3, the performance of the Bayesian framework is benchmarked against a permutation test with *F* = 100 on a synthetic dataset of 10,000 COGs. As it can be readily observed, the posterior probability generated by the Bayesian framework yields a significantly more robust predictor of eggNOG/COG regulation [Area under the curve (AUC): 0.99] than a conventional permutation test p-value (AUC: 0.88).

### Analysis of the copper-homeostasis CsoR regulon in the human gut microbiome

To evaluate the proposed inference method in a real life setting, we analyzed the copper-homeostasis regulon controlled by CsoR in the human gut microbiome. Together with CopY and CueR, CsoR-family members are well-characterized copper-responsive regulators that detect and modulate the abundance of copper ions in the cell [25]. CsoR provides a suitable target for analysis, because it is presumed to be the sole regulator of copper homeostasis in Clostridiales, the second most abundant bacterial order in the IGC MetaHIT project dataset, while being noticeably absent in the most abundant order (Bacteroidales) [17, 26]. We analyzed the CsoR regulon by running the Bayesian inference pipeline on operons containing genes mapping to the Firmicutes. Computation was sped up by adjusting sensitivity with *θ* = 6.65 (6 standard deviations below the CsoR motif mean). This substantially decreased the number of processed promoters while increasing the prior for regulation *P(R)* only to 0.01 (Fig. 2). We established a mean probability of regulation of 0.9 for the set of putatively regulated eggNOG/COGs and required that they had at least 5 promoters mapping to them at the established *θ* value.

The results shown in Table 1 provide an outline of the Firmicutes CsoR meta-regulon of the human gut microbiome. The inferred CsoR meta-regulon is in broad agreement with the reported CsoR regulons in Firmicutes [23, 24, 27, 28], but displays also several characteristic features that have not been previously reported. The inferred human gut Firmicutes CsoR meta-regulon comprises six distinct eggNOG/COG identifiers with annotated function, but is primarily defined by two COG identifiers that encompass 96 % of the putatively CsoR-regulated promoters (Additional files 2, 3). COG1937 maps to the CsoR repressor, and all the putatively regulated complete gene sequences mapping to this COG contain the conserved C-H-C motif (Additional file 4). This indicates that these COG1937 instances are functional copper-responsive regulators and suggests that the reported self-regulation of CsoR is a common trait of human gut Firmicutes species [17, 23]. COG2217 maps to the copper-translocating P-type ATPases (CopA). These proteins harbor heavy metal-associated (HMA; IPR006121), haloacid dehydrogenase-like (HAD-like; IPR023214) and P-type ATPase A (IPR008250) domains and are canonical members of the Firmicutes CsoR regulon [25]. The remaining eggNOG/COGs map to proteins containing a HMA (IPR006121) domain [NOG218972, NOG81268], an unknown function (DUF2318; IPR018758) membrane domain [NOG72602] or HMA (IPR006121), DsbD_2 (IPR003834) and DUF2318 (IPR018758) transmembrane domains [COG2836]. Proteins mapping to NOG218972 and NOG81268 are often annotated as copper chaperones, whereas those mapping to COG2836 are mainly annotated as heavy metal transport/detoxification proteins, and those mapping to NOG72602 are simply annotated as membrane proteins. Analysis of site score distribution for the eggNOG/COGs reported in Table 1 indicates the presence of a single putative false positive. The sequences mapping to NOG109008 belong to clonal instances of a glycoside hydrolase family 18 protein-coding sequence harboring an average (19.42 score) putative CsoR-binding site in its promoter region.

**Table 1:** Inferred human gut Firmicutes CsoR meta-regulon

eggNOG / COG	eggNOG 4.0 annotation	Domains	Mapped operons	Operons for analysis	P(R|D)	Operon with COG1937	Operon with COG2217	Dual sites
COG1937	Transcriptional repressor	IPR003735	530	332	1	332	94	93/221
COG2217	p-type ATPase	IPR006121, IPR023214, IPR008250	1580	422	1	84	422	94/204
NOG218972	Heavy-metal-associated domain	IPR006121	16	7	1	1	2	3/7
NOG72602	Predicted membrane protein	IPR018758	16	5	1	0	0	1/4
NOG109008	N/A	IPR018242	18	5	1	0	0	0/5
COG2836	Membrane protein	IPR006121, IPR003834, IPR018758	36	8	1	0	1	1/3
NOG81268	Heavy-metal-associated domain	IPR006121	34	6	1	0	1	1/5

Operons for analysis denotes the total number of operons mapping to each eggNOG/COG after sensitivity adjustment. P(R|D) designates the posterior probability of regulation for the eggNOG/COG. The Operon with COG1937 and Operon with COG2217 columns indicate the number of genes mapping to an eggNOG/COG that were assigned to an operon containing also COG1937 or COG2217, respectively. Dual sites denotes the number of sequences mapping to an eggNOG/COG harboring two high-confidence sites, out of the total number of sequences mapping to that eggNOG/COG with high-confidence sites

**Fig. 4:**
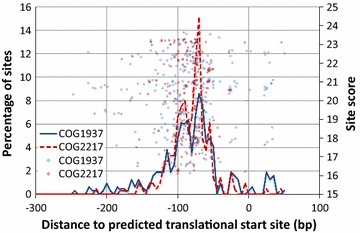
Distribution of site scores and high-confidence sites (sites with scores larger than 16.17) in the promoter region of putatively regulated operons mapping to COG2217 and COG1937

Analysis of the dominating eggNOG/COG identifiers in the human gut Firmicutes CsoR meta-regulon (COG1937 and COG2217) indicates that the copper-responsive regulator and copper-translocating P-type ATPase genes mapping to these regulated COGs are found in an operon configuration in a relatively small fraction of instances (Table 1; Additional file 5). Protein-coding genes mapping to COG2217 are in some cases associated with those coding for chaperone-like proteins (NOG218972, COG2836 and NOG81268), but there is only one instance of a three-gene operon mimicking the CsoR-CopA-CopZ organization described in *Listeria monocytogenes* [27]. The promoter region of protein-coding sequences mapping to COG1937 and COG2217 reveals that around half of them contain high-confidence CsoR-binding sites (sites with score larger than two standard deviations below the mean for the CsoR motif). On both sequence sets, the distribution of high-confidence CsoR-binding sites peaks around 90 and 65 bp upstream of the predicted translation start site (TLS) (Fig. 4). Interestingly, almost half of these promoter sequences contain two high-confidence sites separated by 26, 36–38 or 51 bp (Additional file 6).

## Discussion

### A Bayesian inference pipeline for metagenomics analysis of regulatory networks

The increasing availability of large metagenomics datasets prompts and enables the development of algorithms to interrogate novel aspects of these heterogeneous sequence repositories. Here we formalize and validate a Bayesian inference framework to analyze the composition of transcriptional regulatory networks in metagenomes. Comparative genomics analyses have long established that the study of bacterial regulons benefits significantly from the availability of genomic data. Enrichment in TF-binding sites upstream of orthologous genes provides the means to curb the false positive rate of in silico methods for detecting these regulatory signals and to identify the key components of a regulatory network [29–32]. Leveraging the clusters of orthologous groups defined in the eggNOG database, here we define a conceptually similar approach to analyze bacterial regulons in metagenomic samples. We apply Bayesian inference to compute the probability that an eggNOG/COG is regulated by a TF with a known binding motif. To facilitate computation, the method assumes independence among the scores over a sequence and a normal distribution for site scores, which may be replaced by the exact distribution [33]. Beyond these assumptions, the method relies only on the availability of priors for site density (*α*) and operon regulation *P(R)*, which can be estimated from reference genomes. The method also provides the means to speed up computation by restricting the set of promoter sequences to be analyzed in a principled manner.

Our results on synthetic datasets show that the method performs as expected, assigning higher posterior values to sequences containing better-scoring sites (Fig. 1a) and to eggNOG/COGs with a larger number of sequences containing putative sites mapping to them (Fig. 2a). These results also illustrate some interesting properties of the approach. The assumption of positional independency provides a simple yet effective method to integrate the contribution of multiple sites in a promoter sequence. This is an important component for the analysis of bacterial regulons, since many bacterial transcriptional regulators exploit cooperative binding between multiple sites to modulate their activity at specific promoters [34–37]. Another element to take into account in metagenomics analysis is the presence of multiple instances of a clonal sequence mapping to an eggNOG/COG. These sequences occur frequently in metagenomic datasets and may carry multiple instances of a putative TF-binding site. The explicit modeling of regulated promoters with a mixture distribution results in lower posterior probabilities for such sequence sets (Fig. 1b), minimizing their assessment as false positives. Sequence sets carrying instances of a site with average score, such as the sequences mapping to NOG109008 (Table 1), may still be assigned high posterior probabilities. Given enough sample size, such false positives can be addressed by the introduction of heuristics based on the variance of scores for high-confidence sites in sequences mapping to an eggNOG/COG.

The proposed approach also provides a method to adjust the sensitivity and speed of the analysis by removing sequences with no evidence of regulation. This method is formally integrated within the Bayesian inference framework by the introduction of a score threshold (*θ*) and the corresponding normalization of priors and likelihoods. In combination with taxonomic filtering (i.e. preserving only sequences mapping to the clade of interest), sensitivity adjustment allows users to focus their analysis on those sequences most likely to contribute relevant information on the regulatory system under analysis. Sensitivity adjustment may hence allow detecting evidence of regulation in eggNOG/COGs with a relatively small percentage of putatively regulated sequences (Fig. 2b). This may be advantageous when assessing regulation in large heterogeneous COGs, where only a small subset of the mapping genes are regulated orthologs, but the progressive refinement of orthologous groups in the eggNOG database will soon address such concerns. Moreover, sensitivity adjustment should be used with caution, since it alters the prior for regulation *P(R)* and can therefore complicate the interpretation of results (Fig. 2b). There is no well-established method to determine what constitutes an acceptable prior when reporting posterior probabilities. As a conservative rule of thumb, one may require that the magnitude of the prior (*φ′*) be of the same order as the complement of the average posterior probability to be reported (1 − *φ*). Nonetheless, the adjusted prior should always be clearly stated when reporting adjusted posterior probabilities to facilitate their assessment. As shown in Fig. 3, the Bayesian framework also performs better as a predictor of eggNOG/COG regulation than a more conventional approach based on permutation tests. This is primarily due to the influence of the Bayesian priors on the posterior probability computation, which greatly reduces the chances of generating false positives in non-regulated eggNOG/COGs. Furthermore, the ability to infer regulation without the need for permuted models decreases run-time and provides consistency across multiple runs.

### Analysis of the human gut Firmicutes CsoR meta-regulon

The analysis of the human gut Firmicutes CsoR meta-regulon reported here provides a first glimpse at the genetic organization of this copper homeostasis regulon in its natural setting. The Firmicutes CsoR meta-regulon is dominated by two putatively regulated COGs that map to two major components of the canonical CsoR regulon (*csoR* and *copA*). These two COGs comprise more than 90 % of the putatively CsoR-regulated promoters, suggesting that these two elements are the sole defining features of the CsoR regulon in the Firmicutes species that populate the human gut. The absence of eggNOG/COG identifiers mapping to the third canonical CsoR regulon member (*copZ*) is noteworthy, since the *copZ* gene codes for a copper chaperone that binds copper ions and transfers them to copper ATPases [26, 38]. Members of several putatively regulated eggNOG/COGs harboring a HMA domain (COG2836, NOG218972 and NOG81268; Table 1) appear to be distant orthologs of *B. subtilis* CopZ, and some might therefore function as copper chaperones. However, the COG associated to *B. subtilis* CopZ (COG2608) obtains a very low posterior probability of regulation in our analysis (9.76 · 10^−15^; Additional file 7). BLAST analysis with *B. subtilis* and *Staphylococcus aureus* CopZ against complete genomes reveals that only one (*Clostridium*) of the ten most abundant *Clostridiales* genera in the human gut microbiome encodes a CopZ homolog (Additional file 8). Furthermore, in reference genomes the *Clostridium copZ* homolog is not in the vicinity of *copA*, does not display a putative CsoR-binding site and appears to be associated with an ArsR-family transcriptional regulator, which may be capable of sensing copper [39]. Together, these data convincingly identify CsoR as a transcriptional regulator of copper homeostasis through a canonical CsoR-binding motif in the gut microbiome Firmicutes. Furthermore, they indicate that the CsoR meta-regulon comprises CsoR and a P-type ATPase (CopA), but not a CopZ-type chaperone, and that the contribution of other heavy-metal-associated domain proteins to CsoR-directed copper homeostasis is comparatively small [25]. The absence of *copZ* from bacterial genomes has been noted before [26, 38], and it has been suggested that the short length of this gene may hinder its detection [26]. Our analysis, however, indicates that, even when present, *copZ* is not regulated by CsoR in the gut microbiome Firmicutes.

Beyond identifying and quantifying the components of a transcriptional regulatory network, our results show that metagenomics analysis of bacterial regulons can also shed light into the wiring of the network and the regulatory mode of the transcription factor. In the species where it has been experimentally described, the CsoR regulon displays a notable variety of genetic arrangements, ranging from single *csoR-copA-copZ* and *copZ-csoR-copA* operons in *L. monocytogenes* and *Thermus thermophilus*, to independent regulation of *csoR* and *copZA* operons in *B. subtilis*, *S. aureus* or *Streptomyces lividans* [23, 24, 27, 28]. Our analysis indicates that *CsoR* regulation in human gut Firmicutes follows this broad pattern, with independent regulation of *csoR* and *copA* being the norm and a relatively small fraction of COG1937 and COG2217 instances associated in putative operons. Similarly, experimental reports of CsoR regulated promoters have documented to date CsoR binding to individual binding sites located at distances ranging from −20 to −180 bp upstream of the predicted translational start site of regulated genes [17, 23, 24, 27]. In contrast, our analysis reveals that 44 % of the sequences mapping to regulated COG1937 and COG2217 instances possess two high-scoring sites separated by three well-defined spacing classes (26, 36–38 and 56 bp; Table 1; Additional file 6). There are currently three available structures for CsoR [17, 28, 40], showing CsoR to form either homodimers (*M. tuberculosis*) or tetramers (*S. lividans and T. thermophilus*), based on a three α-helix bundle. However, in the absence of co-crystals and of a canonical DNA-binding fold, the exact mechanism by which CsoR recognizes DNA remains elusive [25, 28]. It has been proposed that CsoR tetramers bind each dyad of the CsoR-binding motif through extensive exposure of DNA to the α1–α2 face of the bundle [28]. In this model the α3 helices of each tetramer may interact and contribute to enhance DNA binding by stabilizing an octameric conformation of CsoR on DNA [41]. Crucially, the ability of α3 helices to interact could be restricted by copper binding, triggering de-repression. Such a model is compatible with the adoption of hexadecameric conformations through extended α3 contacts. In this light, the location of CsoR-binding site relative to the TLS and the spacing distances observed for site pairs in our analysis are reminiscent of promoter architectures that leverage multiple sites to induce DNA bending [34, 35]. This suggests that higher-order conformations of DNA-bound CsoR may be exploited by gut microbiome Firmicutes and other species to fine-tune the cellular response to excess copper ions.

## Conclusions

In this work we introduce and validate a method for the analysis of transcriptional regulatory networks from metagenomic data. By adopting a Bayesian inference framework, our method provides the means to infer regulatory networks from metagenomic data in a systematic and reproducible way, generating posterior probability values that facilitate the interpretation of results. The availability of robust methods for metagenomic regulon inference paves the way for the comparative analysis of regulatory networks across metagenomes, which has the potential to address fundamental questions about the evolution of bacterial regulatory networks. Validation of the method on the CsoR meta-regulon of gut microbiome Firmicutes provides convincing evidence that CsoR is a functional copper-responsive regulator of copper homeostasis in human gut. By virtue of the taxonomic composition of the human gut microbiome, our analysis also constitutes the first description of the CsoR-governed copper homeostasis regulon of a broad taxonomic group, the Clostridiales, encompassing several poorly characterized species of increasing clinical interest. Notable aspects of this putative regulatory network include the absence of CopZ-type copper chaperones and the likely use of dual CsoR-binding sites to fine-tune gene regulation.

## Additional files

10.1186/s13015-016-0082-8 Appendix. Derivation of the soft-max scoring function.
10.1186/s13015-016-0082-8 Set of promoters mapping to putatively regulated eggNOG/COGs after adjusting for sensitivity with *θ* = 6.65. The table reports the eggNOG/COG identifier, the MetaHIT IGC gene identifier, composed of sample and gene identifiers, the gene strand and its promoter region sequence, and the gene and protein sequences.
10.1186/s13015-016-0082-8 Distribution of eggNOG/COG posterior probabilities as a function of the number of promoter sequences mapping to the eggNOG/COG after adjusting for sensitivity with *θ* = 6.65. The x-axis indicates eggNOG/COG rank number, sorted by decreasing posterior probability. Bubble size indicates the number of promoters mapping to a given eggNOG/COG.
10.1186/s13015-016-0082-8 Sequence logo summarizing the multiple sequence alignment of putatively regulated protein sequences mapping to COG1937. Alignment was performed with CLUSTALW in profile alignment mode, using the structural information in the *M. tuberculosis* CsoR P9WP49 UniProtKB entry to define gap penalties. The C-H-C motif residues are denoted by red arrows.
10.1186/s13015-016-0082-8 Putative operons mapping two or more putatively regulated eggNOG/COGs. Gene identifiers in the same row constitute putative operons. The table reports IGC gene identifiers, composed of sample and gene identifiers, the putative regulation and the strand on which the gene has been predicted.
10.1186/s13015-016-0082-8 Distribution of distance between high-confidence sites (bp) for promoters with more than one high-confidence site.
10.1186/s13015-016-0082-8 Posterior probability assigned to eggNOG/COG identifiers after sensitivity adjustment with *θ* = 6.65. The table lists the eggNOG/COG identifier, its eggNOG 4.0 annotation and functional category, the number of mapped promoters before and after sensitivity adjustment and the posterior probability for all eggNOG/COGs with at least 5 promoters mapping to them after sensitivity adjustment.
10.1186/s13015-016-0082-8 Average abundance (%) of the 10 most abundant Clostridiales genera in the 401 MetaHit samples analyzed in this work. The first column indicates the putative presence (+) or absence (−) of a CopZ homolog as determined through independent BLASTP searches using *B. subtilis* and *S. aureus* CopZ protein sequences with a cutoff e-value of 10^−15^. Table compiled from data reported in Li et al. Nature Biotechnology 32, 834–841 (2014).

## Abbreviations

ATPase: adenylpyrophosphatase
BLAST: basic local alignment search tool
COG: clusters of orthologous groups
CsoR: copper-sensitive operon repressor
DUF: domain of unknown function
eggNOG: evolutionary genealogy of genes: non-supervised orthologous groups
HAD-like: haloacid dehydrogenase-like
HMA: heavy metal-associated
ORF: open reading frame
TF: transcription factor
TLS: translation start site
IGC: integrated non-redundant gene catalog
MetaHIT: metagenomics of the human intestinal tract
ROC: receiver-operating characteristic
AUC: area under the curve

## References

[1] Sleator RD, Shortall C, Hill C (2008). Metagenomics. Lett Appl Microbiol.

[2] Venter JC, Remington K, Heidelberg JF, Halpern AL, Rusch D, Eisen JA, Wu D, Paulsen I, Nelson KE, Nelson W, Fouts DE, Levy S, Knap AH, Lomas MW, Nealson K, White O, Peterson J, Hoffman J, Parsons R, Baden-Tillson H, Pfannkoch C, Rogers Y-H, Smith HO (2004). Environmental genome shotgun sequencing of the Sargasso Sea. Science.

[3] Tringe SG, von Mering C, Kobayashi A, Salamov AA, Chen K, Chang HW, Podar M, Short JM, Mathur EJ, Detter JC, Bork P, Hugenholtz P, Rubin EM (2005). Comparative metagenomics of microbial communities. Science.

[4] Ward AC, Bora N (2006). Diversity and biogeography of marine actinobacteria. Curr Opin Microbiol.

[5] Qin J, Li R, Raes J, Arumugam M, Burgdorf KS, Manichanh C, Nielsen T, Pons N, Levenez F, Yamada T, Mende DR, Li J, Xu J, Li S, Li D, Cao J, Wang B, Liang H, Zheng H, Xie Y, Tap J, Lepage P, Bertalan M, Batto JM, Hansen T, Le Paslier D, Linneberg A, Nielsen HB, Pelletier E, Renault P (2010). A human gut microbial gene catalogue established by metagenomic sequencing. Nature.

[6] Hug LA, Beiko RG, Rowe AR, Richardson RE, Edwards EA (2012). Comparative metagenomics of three Dehalococcoides-containing enrichment cultures: the role of the non-dechlorinating community. BMC Genom.

[7] Segata N, Haake SK, Mannon P, Lemon KP, Waldron L, Gevers D, Huttenhower C, Izard J (2012). Composition of the adult digestive tract bacterial microbiome based on seven mouth surfaces, tonsils, throat and stool samples. Genome Biol.

[8] Thomas T, Gilbert J, Meyer F (2012). Metagenomics—a guide from sampling to data analysis. Microb Inf Exp.

[9] De Filippo C, Ramazzotti M, Fontana P, Cavalieri D (2012). Bioinformatic approaches for functional annotation and pathway inference in metagenomics data. Brief Bioinform.

[10] Warnecke F, Luginbühl P, Ivanova N, Ghassemian M, Richardson TH, Stege JT, Cayouette M, McHardy AC, Djordjevic G, Aboushadi N, Sorek R, Tringe SG, Podar M, Martin HG, Kunin V, Dalevi D, Madejska J, Kirton E, Platt D, Szeto E, Salamov A, Barry K, Mikhailova N, Kyrpides NC, Matson EG, Ottesen EA, Zhang X, Hernández M, Murillo C, Acosta LG (2007). Metagenomic and functional analysis of hindgut microbiota of a wood-feeding higher termite. Nature.

[11] Ley RE, Hamady M, Lozupone C, Turnbaugh PJ, Ramey RR, Bircher JS, Schlegel ML, Tucker TA, Schrenzel MD, Knight R, Gordon JI (2008). Evolution of mammals and their gut microbes. Science.

[12] Zheng W, Zhang Z, Liu C, Qiao Y, Zhou D, Qu J, An H, Xiong M, Zhu Z, Zhao X (2015). Metagenomic sequencing reveals altered metabolic pathways in the oral microbiota of sailors during a long sea voyage. Sci Rep.

[13] Tobar-Tosse F, Rodríguez AC, Vélez PE, Zambrano MM, Moreno PA (2013). Exploration of noncoding sequences in metagenomes. PLoS One.

[14] Fernandez L, Mercader JM, Planas-Fèlix M, Torrents D (2014). Adaptation to environmental factors shapes the organization of regulatory regions in microbial communities. BMC Genom.

[15] Cornish JP, Sanchez-Alberola N, O’Neill PK, O’Keefe R, Gheba J, Erill I (2014). Characterization of the SOS meta-regulon in the human gut microbiome. Bioinformatics.

[16] Li J, Jia H, Cai X, Zhong H, Feng Q, Sunagawa S, Arumugam M, Kultima JR, Prifti E, Nielsen T, Juncker AS, Manichanh C, Chen B, Zhang W, Levenez F, Wang J, Xu X, Xiao L, Liang S, Zhang D, Zhang Z, Chen W, Zhao H, Al-Aama JY, Edris S, Yang H, Wang J, Hansen T, Nielsen HB, Brunak S (2014). An integrated catalog of reference genes in the human gut microbiome. Nat Biotechnol.

[17] Liu T, Ramesh A, Ma Z, Ward SK, Zhang L, George GN, Talaat AM, Sacchettini JC, Giedroc DP (2007). CsoR is a novel Mycobacterium tuberculosis copper-sensing transcriptional regulator. Nat Chem Biol.

[18] Powell S, Forslund K, Szklarczyk D, Trachana K, Roth A, Huerta-Cepas J, Gabaldón T, Rattei T, Creevey C, Kuhn M, Jensen LJ, von Mering C, Bork P (2014). eggNOG v4.0: nested orthology inference across 3686 organisms. Nucleic Acids Res.

[19] Novichkov PS, Laikova ON, Novichkova ES, Gelfand, Arkin AP, Dubchak I, Rodionov DA (2010). RegPrecise: a database of curated genomic inferences of transcriptional regulatory interactions in prokaryotes. Nucleic Acids Res.

[20] Kiliç S, White ER, Sagitova DM, Cornish JP, Erill I (2014). CollecTF: a database of experimentally validated transcription factor-binding sites in Bacteria. Nucleic Acids Res.

[21] Buchfink B, Xie C, Huson DH (2015). Fast and sensitive protein alignment using DIAMOND. Nat Methods.

[22] Haverty PM, Hansen U, Weng Z (2004). Computational inference of transcriptional regulatory networks from expression profiling and transcription factor binding site identification. Nucleic Acids Res.

[23] Smaldone GT, Helmann JD (2007). CsoR regulates the copper efflux operon copZA in *Bacillus subtilis*. Microbiology.

[24] Baker J, Sengupta M, Jayaswal RK, Morrissey JA (2011). The *Staphylococcus aureus* CsoR regulates both chromosomal and plasmid-encoded copper resistance mechanisms. Environ Microbiol.

[25] Rademacher C, Masepohl B (2012). Copper-responsive gene regulation in bacteria. Microbiology.

[26] Solioz M, Abicht HK, Mermod M, Mancini S (2010). Response of gram-positive bacteria to copper stress. J Biol Inorg Chem.

[27] Corbett D, Schuler S, Glenn S, Andrew PW, Cavet JS, Roberts IS (2011). The combined actions of the copper-responsive repressor CsoR and copper-metallochaperone CopZ modulate CopA-mediated copper efflux in the intracellular pathogen *Listeria monocytogenes*. Mol Microbiol.

[28] Dwarakanath S, Chaplin AK, Hough MA, Rigali S, Vijgenboom E, Worrall JAR (2012). Response to copper stress in *Streptomyces lividans* extends beyond genes under direct control of a copper-sensitive operon repressor protein (CsoR). J Biol Chem.

[29] Tan K, Moreno-Hagelsieb G, Collado-Vides J, Stormo GD (2001). A comparative genomics approach to prediction of new members of regulons. Genome Res.

[30] Rodionov DA, Mironov AA, Gelfand MS (2002). Conservation of the biotin regulon and the BirA regulatory signal in Eubacteria and Archaea. Genome Res.

[31] Sanchez-Alberola N, Campoy S, Barbe J, Erill I (2012). Analysis of the SOS response of Vibrio and other bacteria with multiple chromosomes. BMC Genom.

[32] GrootKormelink T, Koenders E, Hagemeijer Y, Overmars L, Siezen RJ, de Vos WM, Francke C (2012). Comparative genome analysis of central nitrogen metabolism and its control by GlnR in the class Bacilli. BMC Genom.

[33] Rahmann S, Müller T, Vingron M. On the power of profiles for transcription factor binding site detection. Stat Appl Genet Mol Biol 2003;2:1544–6115. doi:10.2202/1544-6115.1032. 10.2202/1544-6115.1032 16646785

[34] Maddocks SE, Oyston PCF (2008). Structure and function of the LysR-type transcriptional regulator (LTTR) family proteins. Microbiology.

[35] Minchin SD, Busby SJ (2009). Analysis of mechanisms of activation and repression at bacterial promoters. Methods.

[36] Pryor EE, Waligora EA, Xu B, Dellos-Nolan S, Wozniak DJ, Hollis T (2012). The transcription factor AmrZ Utilizes multiple DNA binding modes to recognize activator and repressor sequences of *Pseudomonas aeruginosa* Virulence Genes. PLoS Pathog.

[37] Cournac A, Plumbridge J (2013). DNA looping in prokaryotes: experimental and theoretical approaches. J Bacteriol.

[38] Argüello JM, Raimunda D, Padilla-Benavides T (2013). Mechanisms of copper homeostasis in bacteria. Front Cell Infect Microbiol.

[39] Liu T, Chen X, Ma Z, Shokes J, Hemmingsen L, Scott RA, Giedroc DP (2008). A Cu(I)-sensing ArsR family metal sensor protein with a relaxed metal selectivity profile. Biochemistry (Mosc).

[40] Sakamoto K, Agari Y, Agari K, Kuramitsu S, Shinkai A (2010). Structural and functional characterization of the transcriptional repressor CsoR from *Thermus thermophilus* HB8. Microbiology.

[41] Ma Z, Cowart DM, Scott RA, Giedroc DP (2009). Molecular insights into the metal selectivity of the copper(I)-sensing repressor CsoR from *Bacillus subtilis*. Biochemistry (Mosc).

[42] Hobbs E, Erill I, Pereira T, O’Neill PK (2016). Metagenome regulatory analysis: working release. Zenodo.

