# Supplementary material for: A Bayesian inference method for the analysis of transcriptional regulatory networks in metagenomic data
Source: Algorithms Mol Biol. 2016 Jul 8;11:19. doi: 10.1186/s13015-016-0082-8 (PMC4938975; doi:10.1186/s13015-016-0082-8)
Supplement: Supplementary file 1 — 10.1186/s13015-016-0082-8 Appendix. Derivation of the soft-max scoring function. [file 13015_2016_82_MOESM1_ESM.pdf]

A manuscript for *Algorithms in Molecular Biology*

A Bayesian inference method for the analysis of transcriptional regulatory networks in metagenomic data

Elisabeth T. Hobbs[†], Talmo Pereira[†], Patrick K. O'Neill & Ivan Erill[*]

Department of Biological Sciences, University of Maryland Baltimore County (UMBC), 1000 Hilltop Circle, Baltimore, MD 21250, USA

[*] To whom correspondence should be addressed: Department of Biological Sciences, University of Maryland Baltimore County (UMBC), 1000 Hilltop Circle, Baltimore, MD 21250 (USA). Phone: +1-410-455-2470. Fax: +1-410-455-3875. Email: erill@umbc.edu.

[†] These two authors contributed equally to this work and should be considered co-first authors.

APPENDIX

Derivation of the soft-max scoring function

The contribution to the TF-binding energy of a site at position *i* in a sequence for a given strand *s* is approximated by the PSSM score, which is defined as:

$$PSSM(S_i^s) = \log_2\left(\frac{P(S_i^s \mid PSWM)}{P(S_i^s \mid bckg)}\right) \qquad (1)$$

where *PSWM* denotes the position-specific weight matrix derived from the known TF-binding motif, *bckg* a mononucleotide background model and the likelihoods $P(S_i^s \mid PSWM)$ and $P(S_i^s \mid bckg)$ are computed assuming independence over site positions [1].

Rearranging terms, we have:

$$P(S_i^s \mid PSWM) = 2^{PSSM(S_i^s)} P(S_i^s \mid bckg) \qquad (2)$$

Since TF-binding events in either orientation (forward strand [*f*] and reverse strand [*r*]) are mutually exclusive and exhaustive, we obtain:

$$P(S_i \mid PSWM) = 2^{PSSM(S_i^f)} P(S_i^f \mid bckg) + 2^{PSSM(S_i^r)} P(S_i^r \mid bckg) \qquad (3)$$

We seek to obtain an effective PSSM score ($PSSM(S_i)$) that subsumes the contributions of both binding events, so that:

$$PSSM(S_i) = \log_2\left(\frac{P(S_i \mid PSWM)}{P(S_i \mid bckg)}\right)$$
$$= \log_2\left(\frac{2^{PSSM(S_i^f)} P(S_i^f \mid bckg) + 2^{PSSM(S_i^r)} P(S_i^r \mid bckg)}{P(S_i \mid bckg)}\right) \quad (4)$$

If we assume that the background model is strand independent (i.e. we compute the frequencies of A/T and G/C, instead of individualized for each base), which comes naturally when we scan both strands, then $P(S_i \mid bckg) = P(S_i^f \mid bckg) = P(S_i^r \mid bckg)$ and:

$$PSSM(S_i) = \log_2\left(2^{PSSM(S_i^f)} + 2^{PSSM(S_i^r)}\right) \quad (5)$$

where $PSSM(S_i)$ denotes the combined PSSM score of a site at position i and $PSSM(S_i^f)$ and $PSSM(S_i^r)$ denote the score of the site at position i in the forward and reverse strands, respectively.

References

1. Stormo GD: **DNA binding sites: representation and discovery**. *Bioinforma Oxf Engl* 2000, **16**:16–23.
