# Supplementary material for: A Bayesian inference method for the analysis of transcriptional regulatory networks in metagenomic data
Source: Algorithms Mol Biol. 2016 Jul 8;11:19. doi: 10.1186/s13015-016-0082-8 (PMC4938975; doi:10.1186/s13015-016-0082-8)
Supplement: Supplementary file 3 — 10.1186/s13015-016-0082-8 Distribution of eggNOG/COG posterior probabilities as a function of the number of promoter sequences mapping to the eggNOG/COG after adjusting for sensitivity with θ = 6.65. The x-axis indicates eggNOG/COG rank number, sorted by decreasing posterior probability. Bubble size indicates the number of promoters mapping to a given eggNOG/COG. [file 13015_2016_82_MOESM3_ESM.pdf]

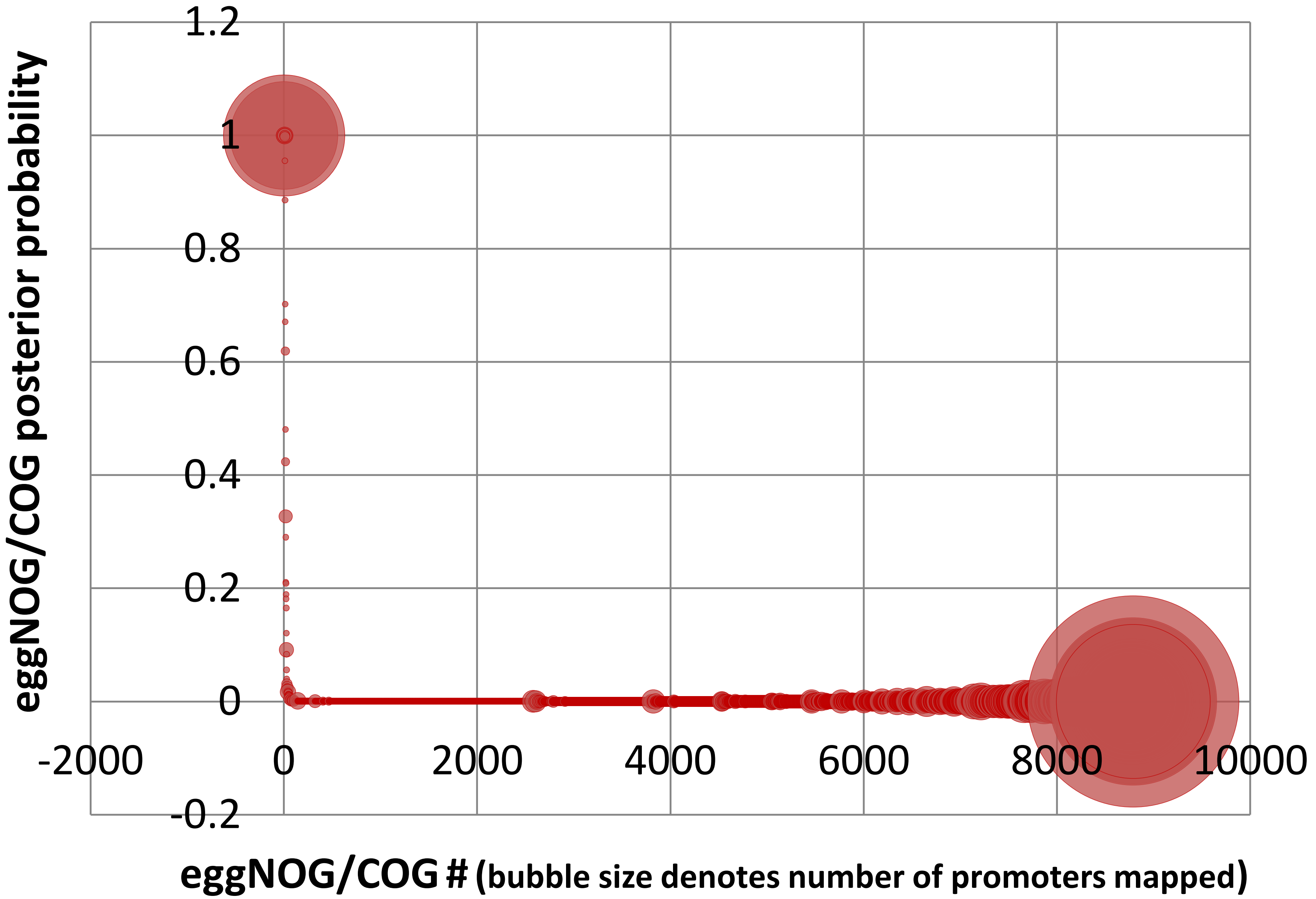

Supplementary file 3 – Distribution of eggNOG/COG posterior probabilities as a function of the number of promoter sequences mapping to the eggNOG/COG after adjusting for sensitivity with ϑ=6.65. The x-axis indicates eggNOG/COG rank number, sorted by decreasing posterior probability. Bubble size indicates the number of promoters mapping to a given eggNOG/COG.
