# Supplementary material for: A Bayesian inference method for the analysis of transcriptional regulatory networks in metagenomic data
Source: Algorithms Mol Biol. 2016 Jul 8;11:19. doi: 10.1186/s13015-016-0082-8 (PMC4938975; doi:10.1186/s13015-016-0082-8)
Supplement: Supplementary file 4 — 10.1186/s13015-016-0082-8 Sequence logo summarizing the multiple sequence alignment of putatively regulated protein sequences mapping to COG1937. Alignment was performed with CLUSTALW in profile alignment mode, using the structural information in the M. tuberculosis CsoR P9WP49 UniProtKB entry to define gap penalties. The C-H-C motif residues are denoted by red arrows. [file 13015_2016_82_MOESM4_ESM.pdf]

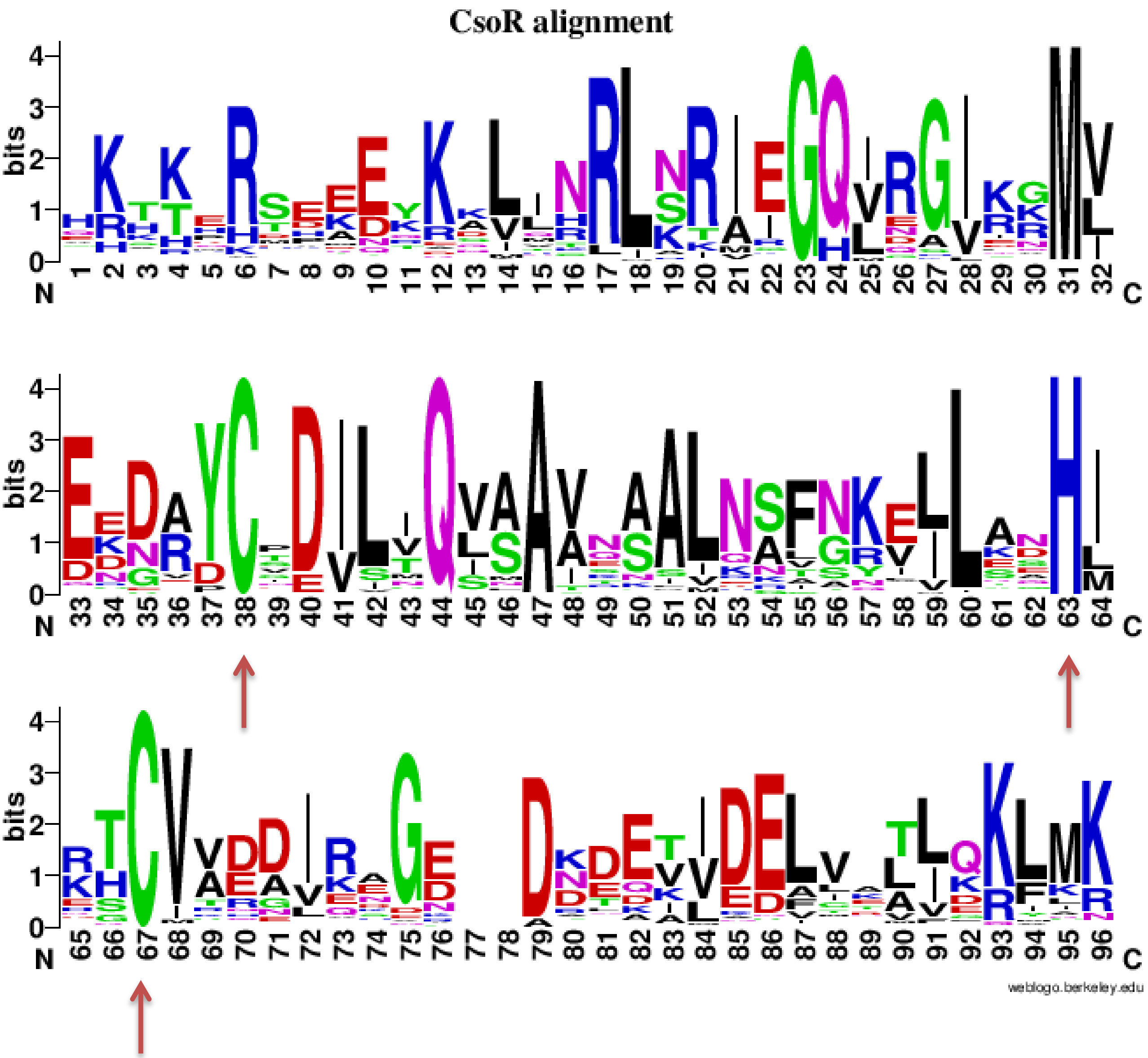

Supplementary file 4 – Sequence logo summarizing the multiple sequence alignment of putatively regulated protein sequences mapping to COG1937. Alignment was performed with CLUSTALW in profile alignment mode, using the structural information in the *M. tuberculosis* CsoR P9WP49 UniProtKB entry to define gap penalties. The C-H-C motif residues are denoted by red arrows.
