# Supplementary material for: A Bayesian inference method for the analysis of transcriptional regulatory networks in metagenomic data
Source: Algorithms Mol Biol. 2016 Jul 8;11:19. doi: 10.1186/s13015-016-0082-8 (PMC4938975; doi:10.1186/s13015-016-0082-8)
Supplement: Supplementary file 6 — 10.1186/s13015-016-0082-8 Distribution of distance between high-confidence sites (bp) for promoters with more than one high-confidence site. [file 13015_2016_82_MOESM6_ESM.pdf]

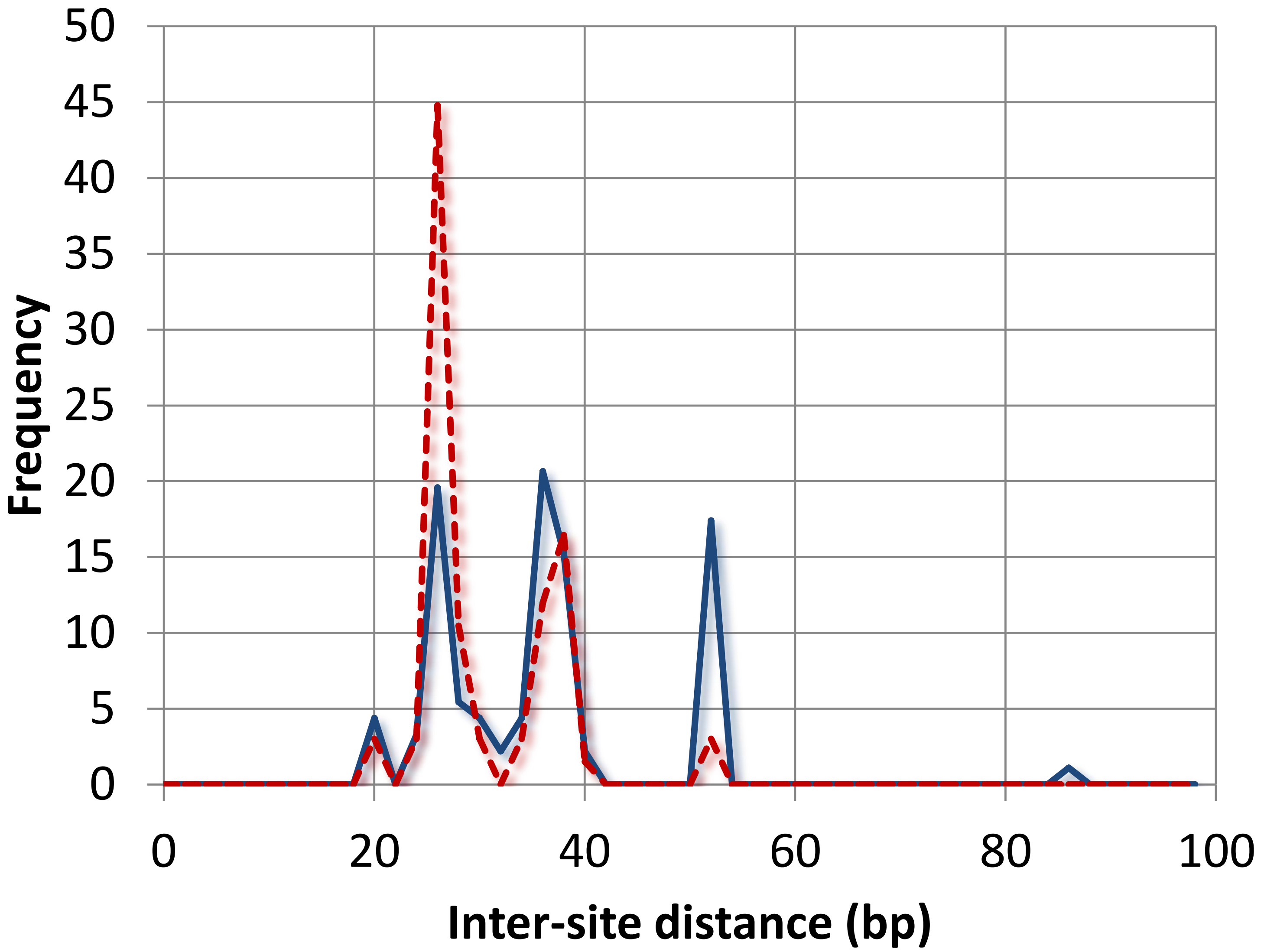

Supplementary file 6 – Distribution of distance between high-confidence sites (bp) for promoters with more than one high-confidence site.
